# Co-emergence of magnetic order and structural fluctuations in magnetite

**DOI:** 10.1038/s41467-019-10949-9

**Published:** 2019-06-28

**Authors:** Giuditta Perversi, Elise Pachoud, James Cumby, Jessica M. Hudspeth, Jon P. Wright, Simon A. J. Kimber, J. Paul Attfield

**Affiliations:** 1 Centre for Science at Extreme Conditions (CSEC) and School of Chemistry, University of Edinburgh, Mayfield Road, Edinburgh, EH9 3JZ UK; 2 European Synchrotron Radiation Facility, BP 156, 38042 Grenoble, France; 3 ICB-Laboratoire Interdisciplinaire Carnot de Bourgogne, Université Bourgogne-Franche Comté, Bâtiment Sciences Mirande, 9 Avenue Alain, 21078 Dijon, France

**Keywords:** Magnetic materials, Magnetic properties and materials, Phase transitions and critical phenomena

## Abstract

The nature of the Verwey transition occurring at *T*_V_ ≈ 125 K in magnetite (Fe_3_O_4_) has been an outstanding problem over many decades. A complex low temperature electronic order was recently discovered and associated structural fluctuations persisting above *T*_V_ are widely reported, but the origin of the underlying correlations and hence of the Verwey transition remains unclear. Here we show that local structural fluctuations in magnetite emerge below the Curie transition at *T*_C_ ≈ 850 K, through X-ray pair distribution function analysis. Around 80% of the low temperature correlations emerge in proportion to magnetization below *T*_C_. This confirms that fluctuations in Fe-Fe bonding arising from magnetic order are the primary electronic instability and hence the origin of the Verwey transition. Such hidden instabilities may be important to other spin-polarised conductors and orbitally degenerate materials.

## Abstract (web summary)

The Verwey transition in magnetite was reported 80 years ago but identifying the underlying mechanism has been difficult. Here the authors show that structural distortions associated with the Verwey transition emerge as local fluctuations at the Curie temperature, confirming their link with magnetic order.

## Introduction

Short-range structural correlations above the Verwey transition^1–4^ were reported in early diffuse scattering studies of magnetite^5^, and their persistence up to at least room temperature has been studied recently^6^ and corroborated by observations of anomalous phonon broadening^7^, a charge gap from optical and photoelectron spectroscopies^8,9^, and magnetic excitations driven by polaronic distortions in resonant inelastic X-ray scattering data^10^. Analysis of the interatomic pair distribution function (PDF)^11^ derived from total X-ray scattering data is a simple method for exploring local structure that is highly sensitive to the displacements of metal atoms associated with the formation of orbital molecules, metal–metal-bonded clusters such as the trimerons observed in magnetite^12^. Persistence of orbital molecules far above their long-range electronic ordering transitions has been discovered from X-ray PDF studies of Li_2_RhO_4_^13^, Li_2_RuO_3_^14^, AlV_2_O_4_^15^, and GaV_2_O_4_^16^.

The thermal variation of local structure in magnetite has been explored here over a wide temperature range encompassing both the Verwey and Curie transitions through synchrotron X-ray PDF analysis.

## Results

### Variable temperature PDF analysis of magnetite

**Fig. 1:**
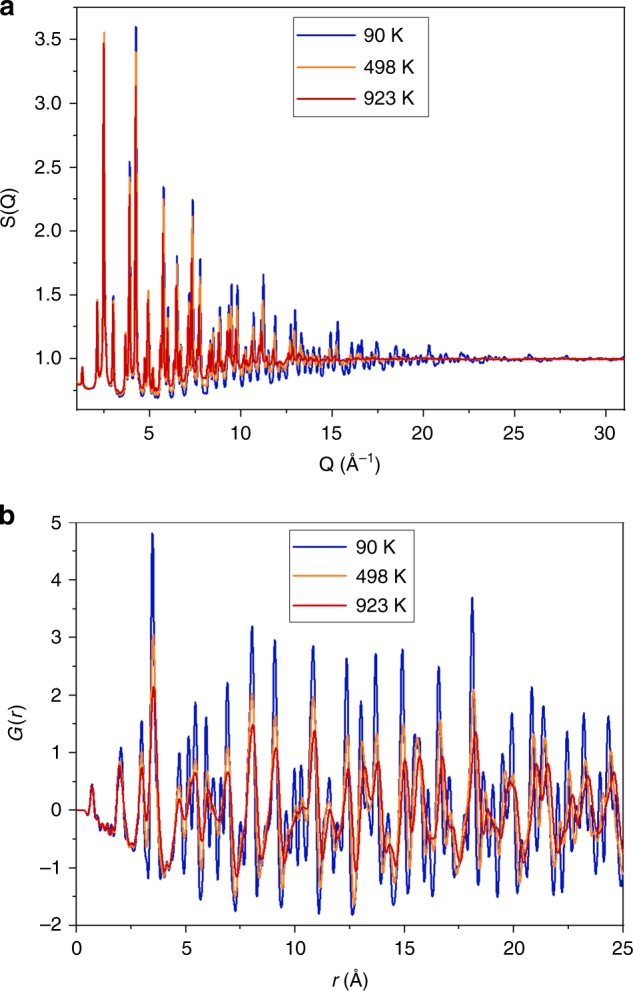
X-ray scattering data for magnetite. **a** Scattering intensities *S*(*Q*) for magnetite at three representative temperatures. **b** Pair distribution functions *G*(*r*) derived from *S*(*Q*) data in **a**. Typical thermal effects leading to loss of high-*Q* features in *S*(*Q*) and broadening of *G*(*r*) peaks are observed

**Fig. 2:**
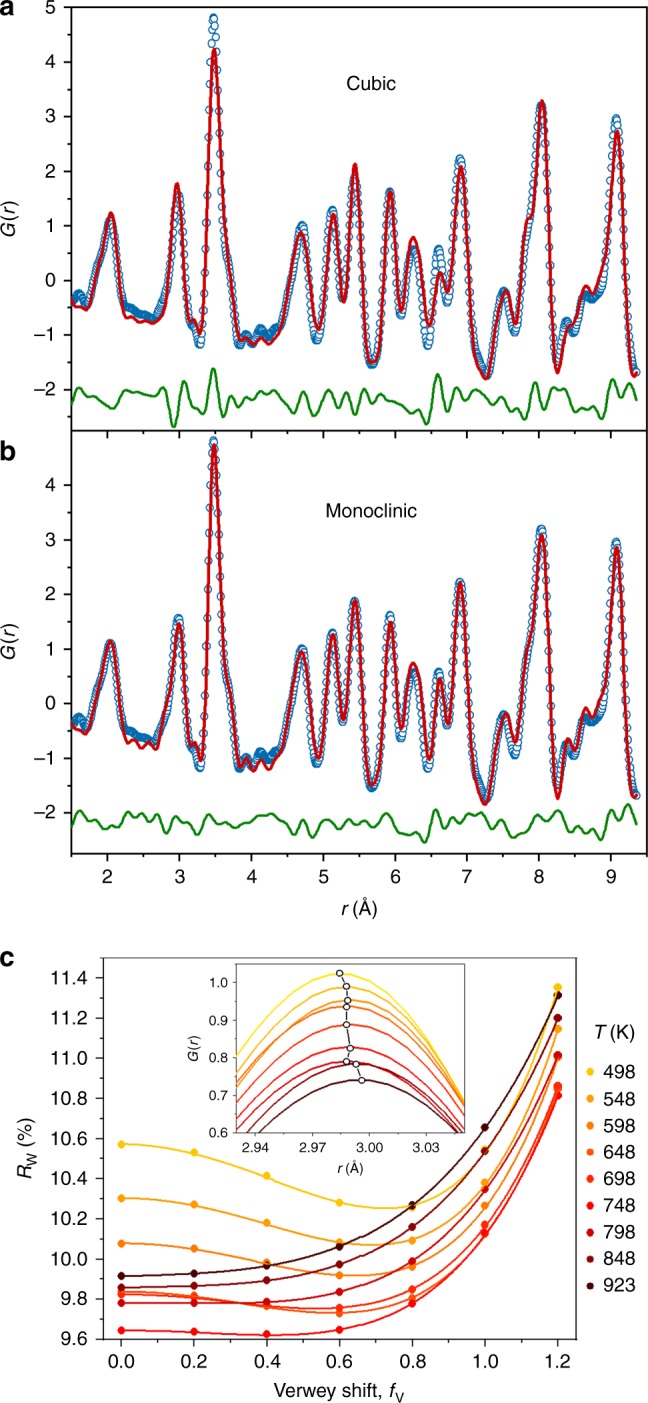
Fitting of magnetite pair distribution functions (PDFs). **a**, **b** Comparison of fits of **a** the cubic (residual *R*w = 15.6%) and **b** the monoclinic superstructure (*R*w = 11.6%) models to the 90 K PDF. The visible improvement of the fit to the *r* ≈ 3 Å peak measuring nearest neighbour Fe–Fe (and O–O) distances and lowering of *R*w demonstrates sensitivity to the monoclinic Verwey superstructure distortions. PDF data are open blue circles, fits are red curves and the difference is below in green. **c** *R*w as a function of the Verwey shift parameter *f*_V_ from fits to the First Unit Cell region of the high-temperature PDFs. The well-defined minimum seen at *f*_V_ ≈ 0.8 for the 498 K fits becomes more shallow and moves to zero at the highest temperatures, as plotted in Fig. 3b. This subtle change of local structure can be seen in the shift in the maximum of the 3 Å peak to slightly longer *r* (inset), consistent with the loss of Fe–Fe distances shortened through trimeron bonding

Experimental details are in the “Methods” section. Total X-ray scattering data from a highly stoichiometric sample of magnetite were recorded from 90 to 923 K. Representative scattering intensity *S*(*Q*) plots and the derived PDFs *G*(*r*) are shown in Fig. 1. The monoclinic superstructure adopted by magnetite below *T*_V_ is very complex with 168 small (<0.24 Å) displacements of atoms from their positions in the high temperature cubic spinel structure^3^. To fit the PDFs over all temperatures, we have used this monoclinic supercell with lattice parameters adjusted to a cubic metric and with each of the 168 atomic coordinates given by *p* = *p*_u_ + *f*_V_(*p*_d_ − *p*_u_) where *p*_u_ is the coordinate from an undistorted high-temperature cubic crystal structure refinement and *p*_d_ is the coordinate in the distorted 90 K structure reported previously^3^. *f*_V_ is a Verwey shift parameter that describes the set of constrained structural displacements, such that *f*_V_ = 0 corresponds to the cubic spinel structure without any local distortions and *f*_V_ = 1 describes the full magnitude of distortions in the 90 K magnetite superstructure^3^. Comparative views of the *f*_V_ = 0 and *f*_V_ = 1 structures are shown in ref. ^4^. Three values of *f*_V_ were determined at each temperature by fitting the structural model to three successive regions of the PDF corresponding approximately to distances from atoms to their neighbours in the same unit cell (First Unit Cell range, covering all interatomic distances for *r* < 9.36 Å) and similarly to atoms in the Second and Third Unit Cell regions. The structural models and fitting procedure are further described in “Methods”, and fits to data and their sensitivity to changing *f*_V_ are shown in Fig. 2.

**Fig. 3:**
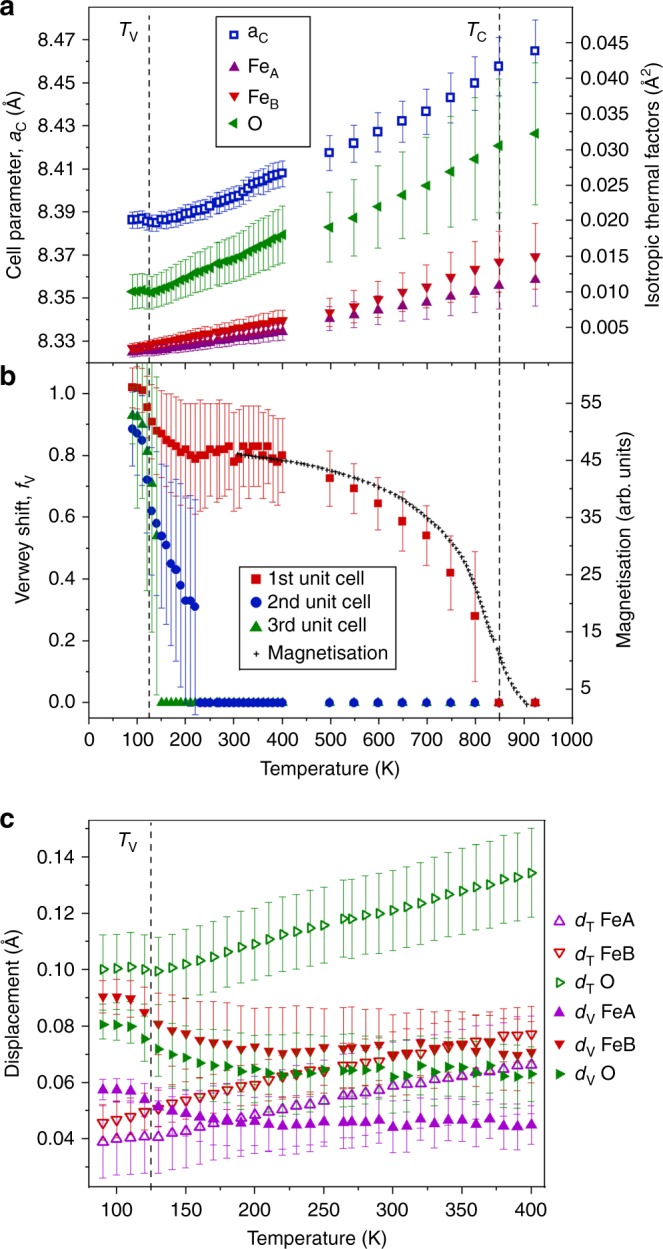
Thermal variations of parameters from magnetite pair distribution function (PDF) fits. **a** Isotropic temperature factors *U*_iso_s for tetrahedral Fe_A_, octahedral Fe_B_, and oxygen sites and the cubic cell parameter from fits to the First Unit Cell PDF range. **b** Verwey shifts *f*_V_ fitted to First, Second and Third Unit Cell PDF ranges. *f*_V_ represents the magnitude of structural distortions due to electronic fluctuations in the cubic phase of magnetite above *T*_V_. The First Unit Cell values show that substantial local structural distortions persist up to *T*_C_ and closely match the variation of bulk magnetisation data taken from ref. ^18^. This demonstrates that the structural and electronic fluctuations responsible for the Verwey transition are a direct result of the long-range magnetic order. **c** Comparison of the atomic displacements due to electronic fluctuations *d*_V_, calculated from atomic coordinates for the First Unit Cell *f*_V_ values, and those due to thermal motion taken as *d*_T_ = √*U*_iso_ from values shown in **a**. Error bars are estimated standard deviations from the refinements

Thermal variations of refined structural quantities from the PDF fits are shown in Fig. 3. The cubic cell parameter and isotropic atomic temperature factors *U*_iso_s in Fig. 3a show a slight anomaly at the Verwey transition (previously found to be *T*_V_ = 123.4 K for this highly pure magnetite sample)^17^ but increase monotonically from 150 up to 923 K. The Verwey shift *f*_V_ measures an averaged local structural distortion due to electronic fluctuations. The thermal variation of *f*_V_ when fitted to the Third Unit Cell separations (between atoms 16.8 to 24.6 Å apart) in Fig. 3b is a typical order-parameter behaviour at a first-order phase transition, with a sharp fall to *f*_V_ = 0 on warming through *T*_V_. Second Unit Cell correlations have a qualitatively similar variation although critical fluctuations decay more gradually above *T*_V_ and are estimated to persist up to 250–300 K. However, structural correlations between atoms in the First Unit Cell range (<9.4 Å apart) show a strikingly different behaviour. *f*_V_ falls a little on warming though the Verwey transition, but around 80% of the structural fluctuations remain in the cubic phase of magnetite up to 500 K. At higher temperatures, *f*_V_ decreases rapidly to zero close to the Curie transition at *T*_C_ ≈ 850 K and so behaves like an order parameter for the magnetic ordering transition with a very similar temperature dependence to the bulk magnetisation reported for a similar synthetic microcrystalline magnetite^18^. This is the key discovery of the present study as it demonstrates that the structural fluctuations responsible for the Verwey transition emerge directly with the long-range magnetic order below the Curie transition and scale with the magnetisation.

The thermal variations of the unit cell parameter and isotropic atomic temperature factors of magnetite plotted in Fig. 3a do not show any discontinuity at *T*_C_ ≈ 850 K, in keeping with previous high-temperature structural studies^19,20^. This demonstrates that our PDF fitting method is robust in decorrelating the overall lattice expansion and phonon motion, quantified by the latter parameters, from the local structural distortions due to electronic fluctuations described by *f*_V_. The magnitudes of the displacements of atoms from their ideal positions due to phonon motion and due to First Unit Cell electronic distortions are compared in Fig. 3c. The electronic distortion displacements are smaller than those due to thermal motion in the disordered high-temperature region, but they increase to a comparable magnitude below *T*_V_. Although the timescale for these structural fluctuations is not directly measured, their lack of correlation with phonon motions suggests that they are essentially static or slowly diffusing. A recent quasi-elastic neutron scattering study of GaV_2_O_4_ spinel above the 415-K charge-ordering transition found that structurally disordered orbital molecules remain well defined and without measurable dynamics up to 1100 K^16^.

### Trimeron fluctuations in cubic magnetite

**Fig. 4:**
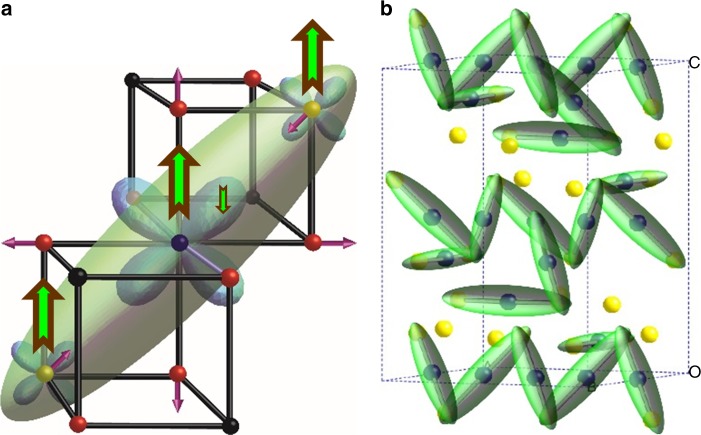
Trimeron bonding driven by magnetic order in magnetite. Charge-ordered Fe^2+^/Fe^3+^ states are shown as blue/yellow spheres, trimerons are green, and oxide ions are red. **a** A single trimeron unit consisting of three Fe sites with parallel *S* = 5/2 spins as shown by the brown–green arrows. Orbital order at the central Fe^2+^ site localises an antiparallel spin electron in one of the *t*_2g_ orbitals, which distorts the local structure through elongation of four Fe–O bonds and shortening of the distances through weak bonding to two Fe neighbours in the same plane, as indicated by the purple arrows. The minority spin electron density is approximated by the ellipsoid shown. **b** Long-range order of trimerons in the monoclinic superstructure formed below the Verwey transition. Corner-sharing of trimerons results in a complex pattern of atomic displacements that has been used to model the local structure in the pair distribution functions

The complex monoclinic superstructure of magnetite below *T*_V_ was previously found to exhibit trimerons (Fig. 4a), small polarons surrounding three Fe units that share corners with each other to build up a long-range ordered network as shown in Fig. 4b. The end-to-end Fe–Fe distance in a trimeron is 6 Å, so their local lattice distortion lies within the First Unit Cell range (< 9.4Å) and hence arises below the Curie transition as demonstrated in Fig. 3b. Persistence of a disordered glassy network of trimerons in the cubic phase of magnetite up to *T*_C_ provides a mechanism for the coupling of the high-temperature structural fluctuations to the magnetism and hence for the origin of the Verwey transition as follows.

Trimerons result from coupled Fe^2+^/Fe^3+^ charge ordering, Fe^2+^ orbital ordering, and weak Fe–Fe bonding effects driven by ferromagnetism within three Fe atom units as described in the caption of Fig. 4a^3^. Comparison with other mixed valent Fe^2+^/Fe^3+^ oxides shows that neither the charge nor orbital orders require long-range spin order, as evidenced by Fe_2_OBO_3_^21,22^ and LuFe_2_O_4_^23^, both of which have charge and orbital ordering transitions at higher temperatures than their magnetic transitions. However, the weak bonding interactions that shorten Fe–Fe distances in the trimerons do require ferromagnetic alignment of the three core *S* = 5/2 spins, as demonstrated recently in CaFe_3_O_5_ where phase-separated trimeron and non-trimeron ground states are observed^24^. Hence, the rapid emergence of structural fluctuations in proportion to magnetisation on cooling below the long-range magnetic ordering temperature confirms that the direct Fe–Fe bonding interactions are the primary driver of the local distortions in magnetite. As suggested previously, the Fe–Fe interactions induce associated charge and orbital fluctuations that become ordered as trimeron units below the Verwey transition^3^. The regular cubic spinel arrangement in which all nearest-neighbour Fe–Fe distances are equivalent is thus unstable with respect to local variations in Fe–Fe separations in the magnetically ordered state of magnetite. There are many degenerate arrangements for linking trimerons with similar connectivities to the observed low-temperature network shown in Fig. 4b^3^, so the long-range electronic order is frustrated. The ratio of energy scales for formation (*T*_C_) and long-range order (*T*_V_) of the electronic distortions serves as a measure of the degree of electronic frustration, analogous to the Weiss to Néel temperature ratio used for frustrated antiferromagnets. The value of *T*_C_/*T*_V_ ≈ 7 confirms that electronic order in magnetite is strongly frustrated.

## Discussion

Analysis of the PDF derived from X-ray scattering data reveals that local structural distortions due to electronic fluctuations emerge at the Curie transition of magnetite. This confirms that fluctuations in Fe–Fe bonding are the dominant electronic instability that couples charge and orbital fluctuations to the magnetic order and hence are the origin of the Verwey transition. Although many experimental studies of magnetite have been carried out at temperatures around *T*_V_, changes near *T*_C_ are much less investigated and will be important to explore the incipient electronic fluctuations further. Such ‘hidden’ local instabilities may also be important to the physics of other spin-polarised conductors and orbitally degenerate materials, and the assumed uniformity of their high temperature structures may require critical reassessment.

## Methods

### Powder X-ray scattering experiments

The same highly pure magnetite powder (Fe_3-*δ*_O_4_ with *δ* < 0.0001) as used in previous studies^3,25–27^ was packed in a 0.5-mm diameter quartz capillary and sealed under Ar atmosphere in a glove bag. Preliminary measurements were performed on beam line ID15B at ESRF, Grenoble and full data were collected on ESRF instrument ID11 with wavelength *λ* = 0.15720(1) Å using a FReLoN camera for diffraction pattern acquisition. A nitrogen cryostream was used to collect patterns between 90 and 400 K in 10 K steps. Small anomalies seen in several refined quantities around 250–300 K in Fig. 3 most likely reflect ice in the sample vicinity leading to additional scattering contributions, as magnetite does not have an intrinsic lattice anomaly in this temperature region. A hot air blower was used to collect data between 498 and 923 K in 50 K steps. Empty capillary data were collected at 90, 200, 473, 573, 673, 773, and 873 K. For each data set, the temperature was stabilised for 10 min and then data were collected for 10 min using the accumulation mode of the detector for 10-s exposure frames. Data were also collected from CeO_2_ and Si standards at 300 K to calibrate the instruments and determine instrumental parameters.

Instrument calibration and image integration was performed with the pyFAI software^28^. The instrument model was recalibrated for every temperature in the cryostat data sets in order to allow for slight detector shifts. A fixed instrument model was used for the hot air blower data. Data sets were converted to scattering intensities *S*(*Q*) as a function of scattering vector *Q* and these were transformed to PDFs *G*(*r*), where *r* is interatomic distance, using the pdfgetx3 suite^29^. Parameters values *Q*_min_ = 1 Å^−1^, *Q*_max_ = 31 Å^−1^, and *r*_poly_ = 0.9 Å were applied to all data sets to enable structural changes in *G*(*r*) to be analysed consistently across the full temperature range. Representative *S*(*Q*) and *G*(*r*) plots are shown in Fig. 1 and Supplementary Fig. 1.

### Structural models for PDF analysis

Structural models used to fit magnetite PDFs at all temperatures were based on the supercell of the low-temperature structure with monoclinic space group *Cc* symmetry, which contains 56 unique atoms with 168 variable coordinates. A cubic model previously refined against 130 K powder X-ray and neutron data^25,26^ was used as the reference undistorted structure. The VESTA software^30^ was used to generate a supercell of the cubic structure with metric *a* = *b* = √2*a*_c_, *c* = 2*a*_c_, *α*  = *β* = *γ* = 90°, where *a*_c_ is the cubic spinel cell parameter, and atomic coordinates *p*_u_ in *Cc* space group symmetry are shown in Supplementary Table 1. This model has *f*_V_ = 0. The previously reported 90 K monoclinic *Cc* structure^3^ was transformed to the same cell metric (from *a* = 11.88881(3) Å, *b* = 11.84940(3) Å, *c* = 16.77515(1) Å, *β* = 90.2363(2)° to *a* = *b* = 11.86182(3) Å, *c* = 16.77515(1) Å, *β* = 90°). Atomic positions were shifted slightly in order to preserve the magnitude of the distortions as generated by the ISODISTORT program^31^. This approximation was found to have little effect on fits of the *Cc* model to the 90 K PDF (*R*w = 11.5% for monoclinic cell parameters vs. *R*w = 11.6% with metric constraints). Coordinates *p*_d_ for this fully distorted reference structure (*f*_V_ = 1) are shown in Supplementary Table 2. To vary the magnitude of the structural distortion, seven structural models were constructed by taking linear combinations of the undistorted and distorted coordinates *p* = *p*_u_ + *f*_V_(*p*_d_ − *p*_u_) for values of the Verwey shift *f*_V_ from 0 to 1.2 in increments of 0.2.

### PDF fits

Refinements of structural models including simulation of termination ripples were performed with the PDFgui software^32^. A fit to the CeO_2_ PDF with a fixed structural model was used to extract the instrument-sensitive parameters *Q*_damp_ = 0.0475(4) Å^−1^ and *Q*_broad_ = 0.0186(3) Å^−1^. It was not possible to minimise the value of *f*_V_ directly in the refinements and so the best-fit value was found by comparing *R*ws for the seven structural models with varying fixed *f*_V_ values.

A ‘box-car’ refinement procedure was followed where, at each temperature, each of the seven structural models were fitted to three regions of the PDF; *r* = 1.50–9.36, 9.30–16.85, and 16.80–24.60 Å, to fit correlations between atoms separated by approximate First, Second, and Third Unit Cell distances, respectively. The cubic cell parameter *a*_c_, separate isotropic thermal parameters *U*_iso_ for tetrahedrally coordinated Fe_A_, octahedral Fe_B_, and O sites, and the peak width correlation parameter δ_1_ were refined during fits to data in the First Unit Cell range. Second and Third Unit Cell fits used *a*_c_ values from the First Unit Cell fits, and *δ*_1_ was set to 0. A further constraint of equal Fe_A_ and Fe_B_ *U*_iso_s was needed to fit Third Unit Cell data sets for temperatures >150 K. Fits to 90 K First Unit Cell data are shown in Fig. 3a.

The best-fit value of *f*_V_ for each temperature and data range was found from the position of the minimum in the *R*w vs. *f*_V_ curve fitted by the arbitrary function:

1 $$Rw = \left( {Af_{\mathrm{V}}^2 + Bf_{\mathrm{V}} + C} \right).{\mathrm{exp}}\left( {Df_{\mathrm{V}}} \right)$$

where *A*, *B*, *C*, and *D* are refined parameters. Fits to *R*w vs. *f*_V_ points are shown in Fig. 2c and Supplementary Fig. 2. Estimated standard deviations (esds) in the best-fit *f*_V_ values were calculated from those in *A*, *B*, *C*, and *D* in Eq. (1). The esds on *f*_V_ values <0.3 are large so these *f*_V_ values were set to zero. The best-fit First Unit Cell’s cell parameters and *U*_iso_s and their errors were calculated by linear interpolation between values at the two *f*_V_ increments closest to the minimum value. Lattice parameters were also fitted to the diffraction data using the Rietveld method and are shown in Supplementary Fig. 3. An approximate correlation length for the structural distortions was calculated from the First Unit Cell *f*_V_ values and is shown in Supplementary Fig. 4.

## Supplementary information

Supplementary Information

## Data Availability

Data that support the findings of this study have been deposited at 10.7488/ds/2574.
