# Supplementary material for: Co-emergence of magnetic order and structural fluctuations in magnetite
Source: Nat Commun. 2019 Jun 28;10:2857. doi: 10.1038/s41467-019-10949-9 (PMC6599026; doi:10.1038/s41467-019-10949-9)
Supplement: Supplementary file 1 — Supplementary Information [file 41467_2019_10949_MOESM1_ESM.pdf]

Supplementary Information

Co-emergence of magnetic order and structural fluctuations in magnetite

Perversi et al

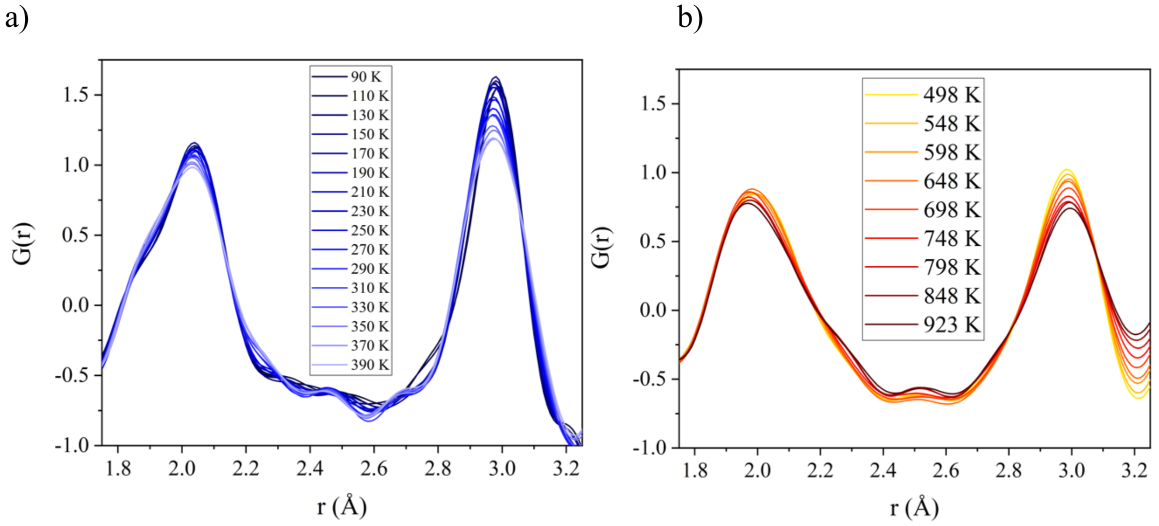

Supplementary Figure 1 – Temperature evolution of the PDFs, displayed in the region of the first and second coordination shells (Fe-O at $r \approx 2.0$ Å and Fe-Fe at $r \approx 3.0$ Å). Small termination error differences between datasets collected using (a) nitrogen and (b) hot-air blower sample environments are apparent in the $r \approx 2.2 – 2.8$ Å region.

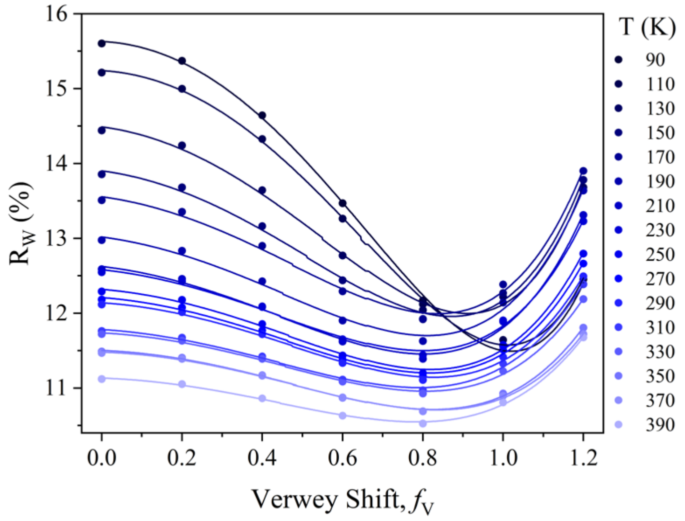

Supplementary Figure 2 –Residual R_w for the First Unit Cell PDF fits plotted as a function of Verwey shift f_V, at 90-300 K. Temperatures are colour coded as indicated by the legend. The discontinuity between 110 and 130 K curves reflects the change from long range to local structural distortions on passing through the Verwey transition at 123 K. The corresponding plot for high temperature fits is in Fig. 2b. The fitting function is Equation (1) in Methods.

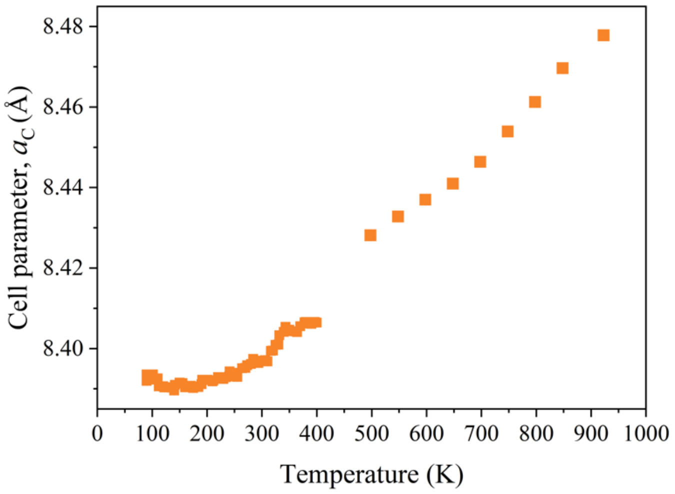

Supplementary Figure 3 – Plot of the cubic lattice parameter fitted to integrated diffraction patterns by Rietveld refinement using the FullProf suite.[1] A cubic spinel model was fitted at all temperatures as it was not possible to refine the monoclinic model below T_V.

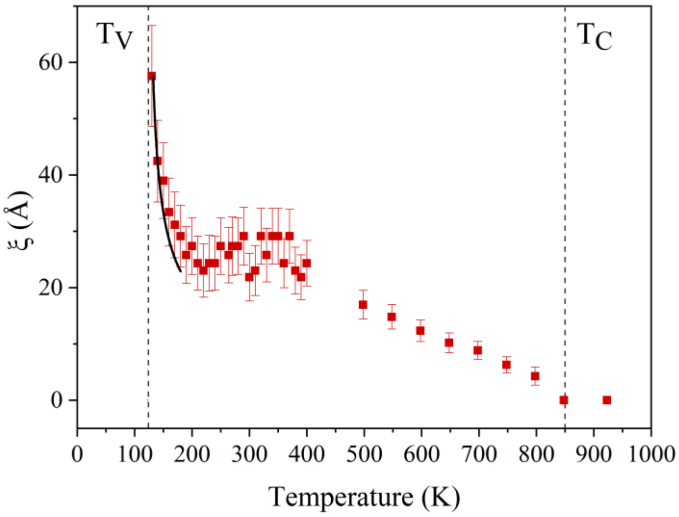

Supplementary Figure 4 – Temperature evolution of an effective correlation length ξ for the local structural distortions in magnetite, derived from the First Unit Cell f_V values through the relation $f_V = e^{-r1/\xi}$ where $r1$ = 5.43 Å is the average interatomic distance in the First Unit Cell range. A critical fit $\xi \sim (T - T_V)^{-\nu}$ is shown for exponent $\nu = 0.5$ in a mean field model. The experimental values deviate above this limit due to the magnetically induced structural correlations that persist up to T_C. Error bars are derived from estimated standard deviations in the refinements.

Supplementary Table 1. Atomic coordinates p_u for the reference undistorted magnetite structure corresponding to Verwey shift $f_V = 0$. The space group is monoclinic *Cc* and lattice parameters $a = b = \sqrt{2}a_c$, $c = 2a_c$, $\alpha = \beta = \gamma = 90°$ are metrically constrained by cubic spinel cell parameter $a_c \approx 8.4$ Å. Coordinates and their estimated standard deviations shown in parentheses are transformed from a refined model of the cubic structure at 130 K as described in Methods. Atom labels are for tetrahedral Fe_A, octahedral Fe_B, and O sites. All sites are fully occupied.

**Atom**	*x*	*y*	*z*
A11	0.627	0	0.8135
A12	0.127	0	0.8135
A13	0.877	0.25	0.0635
A14	0.877	0.25	0.5635
A21	0.377	0	0.6885
A22	0.877	0	0.6885
A23	0.627	0.25	0.9385
A24	0.627	0.25	0.4385
B1A1	0.002	0	0.001
B1A2	0.502	0	0.001
B1B1	0.252	0.25	0.251
B1B2	0.252	0.25	0.751
B2A1	0.127	0.125	0.626
B2A2	0.127	0.125	0.126
B2B1	0.627	0.125	0.626
B2B2	0.627	0.125	0.126
B31	0.377	0.375	0.876
B32	0.377	0.375	0.376
B33	0.877	0.375	0.876
B34	0.877	0.375	0.376
B41	0.252	0	0.001
B42	0.752	0	0.001
B43	0.502	0.25	0.251
B44	0.502	0.25	0.751
O11	0.75690(3)	0.00000(3)	0.87845(15)
O12	0.25690(3)	0.00000(3)	0.87845(15)
O13	0.00690(3)	0.25000(3)	0.12845(15)
O14	0.00690(3)	0.25000(3)	0.62845(15)
O21	0.12700(3)	0.37010(3)	0.74855(15)
O22	0.12700(3)	0.37010(3)	0.24855(15)
O23	0.62700(3)	0.37010(3)	0.74855(15)
O24	0.62700(3)	0.37010(3)	0.24855(15)
O31	0.87700(3)	0.12010(3)	0.99855(15)
O32	0.87700(3)	0.12010(3)	0.49855(15)
O33	0.37700(3)	0.12010(3)	0.99855(15)
O34	0.37700(3)	0.12010(3)	0.49855(15)
O41	0.49710(3)	0.00000(3)	0.87845(15)
O42	0.99710(3)	0.00000(3)	0.87845(15)
O43	0.74710(3)	0.25000(3)	0.12845(15)
O44	0.74710(3)	0.25000(3)	0.62845(15)
O5A1	0.24710(3)	0.00000(3)	0.62355(15)
O5A2	0.74710(3)	0.00000(3)	0.62355(15)
O5A3	0.49710(3)	0.25000(3)	0.87355(15)
O5A4	0.49710(3)	0.25000(3)	0.37355(15)
O5B1	0.62700(3)	0.37990(3)	0.00345(15)
O5B2	0.62700(3)	0.37990(3)	0.50345(15)
O5B3	0.12700(3)	0.37990(3)	0.00345(15)
O5B4	0.12700(3)	0.37990(3)	0.50345(15)
O6A1	0.37700(3)	0.12990(3)	0.25345(15)
O6A2	0.37700(3)	0.12990(3)	0.75345(15)
O6A3	0.87700(3)	0.12990(3)	0.25345(15)
O6A4	0.87700(3)	0.12990(3)	0.75345(15)
O6B1	0.00690(3)	0.00000(3)	0.62355(15)
O6B2	0.50690(3)	0.00000(3)	0.62355(15)
O6B3	0.25690(3)	0.25000(3)	0.87355(15)
O6B4	0.25690(3)	0.25000(3)	0.37355(15)

Supplementary Table 2. Atomic coordinates p_d for the reference fully distorted magnetite structure corresponding to Verwey shift f_V = 1. The space group is monoclinic *Cc* and lattice parameters $a = b = \sqrt{2}a_c$, $c = 2a_c$, $\alpha = \beta = \gamma = 90°$ are metrically constrained by cubic spinel cell parameter $a_c \approx 8.4$ Å. Coordinates and their estimated standard deviations shown in parentheses are transformed from a refined model of the monoclinic structure at 90 K as described in Methods. Atom labels are for tetrahedral Fe_A, octahedral Fe_B, and O sites. All sites are fully occupied.

Atom	*x*	*y*	*z*
A11	0.62943(2)	0.00502(2)	0.81268(13)
A12	0.12546(2)	0.00162(2)	0.81099(13)
A13	0.87529(2)	0.24867(2)	0.06528(13)
A14	0.87834(2)	0.25204(2)	0.56495(13)
A21	0.37481(18)	0.00477(2)	0.69019(11)
A22	0.88092(18)	0.00074(2)	0.68841(11)
A23	0.62680(19)	0.25398(2)	0.93773(10)
A24	0.62495(18)	0.24724(19)	0.43778(11)
B1A1	-0.00237(3)	0.00076(3)	-0.00317(14)
B1A2	0.50207(3)	0.00050(3)	0.00160(14)
B1B1	0.25949(3)	0.24780(3)	0.25357(15)
B1B2	0.24778(2)	0.25639(2)	0.75250(13)
B2A1	0.12586(2)	0.12776(15)	0.62574(14)
B2A2	0.12898(2)	0.12538(16)	0.12301(13)
B2B1	0.62808(3)	0.12601(10)	0.62642(18)
B2B2	0.62683(3)	0.11338(10)	0.12168(17)
B31	0.37714(2)	0.37945(17)	0.87971(13)
B32	0.37563(2)	0.36913(16)	0.37427(13)
B33	0.87664(2)	0.38747(15)	0.88065(12)
B34	0.87619(2)	0.37518(18)	0.37661(13)
B41	0.25136(2)	0.00135(2)	0.00100(14)
B42	0.75077(2)	0.00212(2)	0.00217(15)
B43	0.50275(2)	0.24372(2)	0.25178(15)
B44	0.50234(3)	0.25412(3)	0.75181(16)
O11	0.75982(12)	0.00477(12)	0.87660(8)
O12	0.25259(12)	0.00327(11)	0.87642(8)
O13	0.00537(13)	0.24490(11)	0.13070(8)
O14	0.01026(13)	0.25207(12)	0.62899(8)
O21	0.12962(13)	0.37691(12)	0.74564(8)
O22	0.12728(13)	0.36320(13)	0.24915(9)
O23	0.62512(12)	0.36980(11)	0.74557(8)
O24	0.62958(13)	0.36784(11)	0.24671(8)
O31	0.87601(12)	0.11957(10)	1.00004(8)
O32	0.87718(13)	0.12378(11)	0.49993(8)
O33	0.37627(12)	0.11928(11)	0.99826(7)
O34	0.37656(12)	0.12353(10)	0.49962(7)
O41	0.50088(11)	0.00176(10)	0.87730(8)
O42	0.99736(12)	0.00223(11)	0.87619(8)
O43	0.74370(13)	0.24950(11)	0.13020(8)
O44	0.74874(12)	0.25644(10)	0.63025(7)
O5A1	0.24335(12)	0.00275(11)	0.62571(7)
O5A2	0.75123(13)	-0.00040(11)	0.62466(7)
O5A3	0.49746(13)	0.25561(12)	0.87114(8)
O5A4	0.49505(13)	0.24641(11)	0.37207(8)
O5B1	0.62576(12)	0.38280(11)	0.00569(8)
O5B2	0.62372(13)	0.37670(12)	0.50330(8)
O5B3	0.12711(13)	0.38186(11)	0.00490(7)
O5B4	0.12595(12)	0.37526(11)	0.50168(7)
O6A1	0.37440(11)	0.12398(11)	0.25502(7)
O6A2	0.37752(11)	0.13749(12)	0.75466(7)
O6A3	0.87800(11)	0.13034(13)	0.25258(7)
O6A4	0.87821(11)	0.13169(11)	0.75170(6)
O6B1	0.01119(11)	0.00229(12)	0.62362(7)
O6B2	0.50298(11)	0.00130(13)	0.62598(7)
O6B3	0.25291(11)	0.25518(13)	0.87214(7)
O6B4	0.25606(11)	0.24263(13)	0.37410(7)

Supplementary References

[1] Rodríguez-Carvajal, J., Recent advances in magnetic structure determination by neutron powder diffraction. *Physica B* **192**, 55-69 (1993).
